# Calcaneocuboid arthrodesis for recurrent clubfeet: what is the outcome at 17-year follow-up?

**DOI:** 10.1007/s11832-014-0557-4

**Published:** 2014-02-07

**Authors:** Alice Chu, Sonia Chaudhry, Debra A. Sala, Dan Atar, Wallace B. Lehman

**Affiliations:** 1 New York Ponseti Clubfoot Center, Pediatric Orthopaedic Surgery, New York University Langone Medical Center, New York University Hospital for Joint Diseases, 301 East 17th street, 10003 New York, NY USA; 2 Department of Orthopaedic Surgery, Soroka Medical Center, Ben Gurion University, Beer Sheva, Israel

**Keywords:** Recurrent clubfoot, Calcaneocuboid arthrodesis, Dillwyn Evans procedure, Clubfoot, Arthrodesis, Fusion

## Abstract

**Purpose:**

Calcaneocuboid arthrodesis was used during revision clubfoot surgery in order to maintain midfoot correction. The purposes of this study were to determine: (1) functional level at 17-year follow-up compared to 5-year follow-up; (2) patients’ current functional level, satisfaction, and pain; and (3) current arthropometric measurements.

**Methods:**

Twenty patients (27 clubfeet) with clubfoot relapse underwent revision soft tissue release and calcaneocuboid fusion between 1991 and 1994. They were previously evaluated at a mean follow-up of 5.5 years. Ten out of 20 patients (13 clubfeet), mean age of 24 years, were reevaluated at mean follow-up of 17.5 years. The Hospital for Joint Diseases Functional Rating System (HJD FRS) for clubfoot surgery, Outcome Evaluation in Clubfoot developed by the International Clubfoot Study Group, the Clubfoot Disease-Specific Instrument, American Academy of Orthopaedic Surgeons (AAOS) Foot and Ankle Outcomes Questionnaire, Laaveg and Ponseti’s functional rating system for clubfoot and pain scale were completed by patient and/or surgeon to assess function, patient satisfaction and pain. Foot and ankle radiographs and anthropometric measurements were reviewed. For HJD FRS, scores from original follow-up were compared to current ones.

**Results:**

The HJD FRS score of all feet was 65.9, demonstrating a significant decline from the original mean score of 77.8 (*p* = 0.03). Excellent/good HJD FRS scores went from 85 to 38 %. Mean AAOS Foot Ankle Outcomes Questionnaire standardized core and shoe comfort scores were 84.6 and 84.5, respectively. Average foot pain was 1.8 on a scale of 1–10. Patients were very/somewhat satisfied with status of foot in 76 % of feet and appearance of foot in 46 % of feet, based on Clubfoot Disease-Specific Instrument questions.

**Conclusions:**

Revision clubfoot surgery with calcaneocuboid fusion in patients 5–8 years of age showed an expected decline in functional outcome measures over a 17-year follow-up period. It still produced comparable results to other studies for a similar population of difficult, revision cases, and should have a place in current surgical treatment techniques.

## Introduction

Stiff, recurrent clubfeet occurring after failed prior treatment is a challenging surgical problem. Over the past six decades, many different procedures have been proposed: revision posteromedial release, osteotomies, external fixation, all of which can be supplemented with fusions around the talus. Most of these are difficult surgeries. The choice of technique is often based on individual preference, since there has never been a published prospective, comparative study.

In 1961, Dillwyn Evans [1] described a procedure consisting of a posteromedial clubfoot release with concomitant calcaneocuboid fusion. The Dillwyn Evans procedure has at least two distinct points that make it relevant to the question of how best to treat stiff clubfeet: it emphasizes correction of the calcaneocuboid joint, and it makes use of arthrodesis to guide foot growth in the skeletally immature child. There are four publications detailing the outcomes of his original patient cohort plus others done under his supervision, with follow-ups ranging from 4 to 23 years [1–4]. Other authors utilized the same procedure, but expanded the indications slightly to include clubfoot patients who had failed prior surgery [5–7]. Follow-ups of these series range from 44 months to almost 10 years [5, 6].

The pendulum for clubfoot treatment has swung substantially from operative to non-operative techniques over the past few decades [8]. In the present climate, the use of revision posteromedial release with calcaneocuboid fusions in this age group (4–8 years) may be unusual. However, the idea of limited fusions has been tested before as one possible solution for the difficult situation of a relapsed clubfoot [1, 9]. In 1999, our institution published on a series of 20 patients (27 feet) treated with the Dillwyn Evans procedure as a salvage procedure after failed initial soft tissue clubfoot releases [7]. At the time of final follow-up, most patients were not yet skeletally mature. The purposes of the present study were to determine: (1) functional level at 17-year follow-up compared to 5-year follow-up; (2) patients’ current functional level, satisfaction, and pain; and (3) current arthropometric measurements.

## Methods

The original cohort consisted of 20 patients (27 feet) who underwent the Dillwyn Evans procedure at a mean age of 6.2 years (range 4.1–9.2). Their first surgery, performed at a mean age of 0.6 years (range 0.3–1.2), was a soft tissue clubfoot release in 21 feet (78 %), a tendo-Achilles lengthening in five feet (19 %) and a tendo-Achilles lengthening/medial capsulotomy in one foot (4 %). At a mean follow-up of 5.5 years (range 2.1–14.7), eight feet (30 %) were rated as excellent, 14 good (52 %), four fair (15 %) and one poor (4 %) on the Hospital for Joint Diseases Functional Rating System (HJD FRS) for clubfoot surgery, which has scores from 0 to 100, with higher scores indicating a better outcome [7].

For the current institutional review board (IRB)-approved study, ten of the 20 patients (50 %) (13 feet) returned for follow-up. Evaluation included clinical examination, radiographs, self-report of functional abilities and pain assessment. The HJD FRS, which assesses all these areas, was repeated for comparison to the original study’s scores [7]. Clinical findings and radiographs (standing anterior-posterior (AP) and lateral of foot, standing AP and lateral of ankle) were scored using the Outcome Evaluation in Clubfoot developed by the International Clubfoot Study Group (ICFSG), which ranges from 0 to 60 points, with a lower score indicating a better result [10]. Twelve points are dedicated to morphology, 36 to functional evaluation and 12 to radiologic evaluation.

Patients completed: (1) the Clubfoot Disease-Specific Instrument (DSI) [11, 12]; (2) American Academy of Orthopaedic Surgeons (AAOS) Foot and Ankle Outcomes Questionnaire with responses referenced to the worse foot in bilateral cases, and standardized scores are reported for two subscales, Core Scale (function and pain) and Shoe Comfort Scale; and (3) three of six questions on Laaveg and Ponseti’s functional rating system for club foot [13]. The physician scored the remaining three clinical questions on the latter questionnaire. Scores on all three of these questionnaires can range from 0 to 100, with a higher score indicating a better outcome.

A pain scale with 1 representing no pain and 10 the worst possible pain was presented verbally, with the intent that it could be presented over phone if necessary. Bilateral leg length, calf circumference, foot length and width, and maximum passive dorsiflexion were measured.

### Data analysis

The mean and range were determined for age at latest follow-up, first surgery and re-do surgery, length of follow-up, anthropometric measurements, and maximum dorsiflexion. The *t* test for related samples was used to compare the HJD FRS scores from the original and current studies [7]. The mean, standard deviation and range were calculated for the ICFSG Outcome Evaluation in Clubfoot [10], AAOS Foot and Ankle Outcomes Questionnaire, Laaveg and Ponseti’s functional rating system [13], DSI [11, 12], and pain scale. The frequency and percentage were determined for the types of first surgery, each classification using the authors’ definitions for the HJD FRS, ICFSG Outcome Evaluation in Clubfoot and Laaveg and Ponseti’s functional rating system, as well as the level of patient satisfaction from the Laaveg and Ponseti’s functional rating system and DSI.

## Results

An attempt was made to contact all of the original 20 patients; however, ten did not have current contact information available. The remaining ten patients (13 feet) with a mean age of 24 years (range 23–26) returned for a clinic visit and radiographs at a mean follow-up of 17.5 years (range 16–19). All were males who were ambulatory without assistance. None had any other definitive surgeries. The first surgery, performed at mean age of 0.6 years (range 0.4–1.0), was soft tissue clubfoot release in 12 of the 13 feet (92 %) and tendo-Achilles/medial capsulotomy in one (8 %). The re-do surgery was performed at a mean age of 6.9 years (5.1–8.2). Four patients had bilateral clubfeet, with three patients having the studied re-do surgery on both feet. The calcaneocuboid joint was observed to be fused radiographically in 11 of the 13 feet. The two unfused feet occurred in one foot of two of the bilateral cases.

For all 13 feet, the current HJD FRS score decreased significantly compared to the original mean score, 65.9 vs 77.8 (*p* = 0.03). At original follow-up, four (31 %) were rated as excellent, seven (54 %) good, and two (15 %) fair. At current follow-up, two (15 %) were rated as excellent, three (23 %) good, four (31 %) fair and four (31 %) poor (Table 1). For the 11 fused feet, the current HJD FRS score was not statistically significantly different than the original score, 65.6 vs 77.7 (*p* = 0.053). At original follow-up, three (27 %) were rated as excellent, seven (63 %) good, and one (9 %) fair. At current follow-up, two (18 %) were rated as excellent, two (18 %) good, three (27 %) fair and four (36 %) poor. One of the two non-fused went from excellent to good and the other remained fair (Table 1).

**Table 1:** HJD Functional Rating Scale: original and current scores

Patient #/side	Original		Current	
	Total	Rating	Total	Rating
1 R	78	Good	36	Poor
2 R	75	Good	46	Poor
3 R	73	Good	51	Poor
L	73	Good	61	Fair
4 R	89	Excellent	95	Excellent
5 R	90	Excellent	77	Good
L (NF)	90	Excellent	70	Good
6 R (NF)	66	Fair	66	Fair
L	66	Fair	79	Good
7 L	71	Good	56	Poor
8 R	75	Good	95	Excellent
9 L	80	Good	60	Fair
10 R	85	Excellent	65	Fair

*NF* not fused

Table 2 contains both scores for all 13 feet and for the 11 fused feet for the ICFSG Outcome Evaluation in Clubfoot, Laaveg and Ponseti’s functional rating system, DSI and pain scale. Using ICFSG Outcome Evaluation in Clubfoot total score [10] for all 13 feet, five (38 %) were good and eight (62 %) were fair. For the 11 fused feet, five (45 %) were good and six (55 %) were fair. For all 13 feet, mean total score was 17.8 (range 10–30). For the 11 fused feet, mean total score was 16.7 (range 10–21). On the AAOS Foot and Ankle Outcomes Questionnaire (only scores for all 13 feet because only worse foot was rated in bilateral cases), the mean standardized scores were 84.6 (range 44–100) for the Core Scale and 84.5 (range 25–100) for the Shoe Comfort Scale. For the Laaveg and Ponseti’s functional rating system [13], the mean score for all 13 feet was 69.3 (range 36–92). One (8 %) was excellent, five (38 %) were good, two (15 %) fair and five (38 %) poor. For the 11 fused feet, the mean score was 70.1 (range 36–92), with one (9 %) excellent, four (36 %) good, two (18 %) fair and four (36 %) poor. For the DSI [11, 12], the mean score for all 13 feet was 63.3 (range 33.3–96.7) and for the 11 fused feet it was 65.5 (range 33.3–96.7).

**Table 2:** Questionnaire scores for all 13 feet and for 11 fused feet

Questionnaire	All 13 feet			11 fused feet		
	Mean	SD	Range	Mean	SD	Range
ICFSG Outcome Evaluation						
Morphology (max 12)	2.5	2.5	0–7	2.0	2.3	0–7
Function (max 36)	11.1	4.0	4–16	11.0	3.9	4–16
Radiology (max 12)	4.2	2.9	0–10	3.8	3.0	0–10
Total (max 60)	17.8	4.9	10–30	16.7	3.6	10–21
Foot and Ankle Questionnaire						
Core	84.6	17.6	44–100			
Shoe Comfort	84.5	26.7	25–100			
Functional rating system	69.3	19.2	36–92	70.1	19.2	36–92
DSI	63.3	19.6	33.3–96.7	65.5	19.6	33.3–96.7
Pain scale	1.8	1.9	1.0–8.0	1.2	0.4	1.0–2.0

Two questionnaires carried patient satisfaction queries. On the Laaveg and Ponseti’s functional rating system [13], patients were very satisfied/satisfied with the end result for eight out of 13 feet (61 %). For the 11 fused feet, seven (63 %) were very satisfied/satisfied. On the DSI [11, 12], in response to a question about the status of the foot, ten of 13 feet (76 %) were scored as very satisfied/somewhat satisfied; for the 11 fused feet, nine (81 %) were very satisfied/somewhat satisfied. Another question on the DSI assessed satisfaction with appearance of the foot; for all 13 feet, six (46 %) were very satisfied/somewhat satisfied; for the 11 fused feet, five (45 %) were very satisfied/somewhat satisfied (Table 3).

**Table 3:** Patient satisfaction, *n* (%)

Satisfaction with	Very satisfied	Satisfied	Neither satisfied nor unsatisfied	Unsatisfied	Very unsatisfied
End result^a^					
13 feet	5 (38)	3 (23)	3 (23)	2 (15)	0
11 feet	5 (45)	2 (18)	2 (18)	2 (18)	0

	Very satisfied	Somewhat satisfied	Somewhat dissatisfied	Very dissatisfied
Status of foot^b^				
13 feet	5 (38)	5 (38)	2 (15)	1 (8)
11 feet	4 (36)	5 (45)	1 (9)	1 (9)
Appearance of foot^b^				
13 feet	4 (31)	2 (15)	5 (38)	2 (15)
11 feet	4 (36)	1 (9)	5 (45)	1 (9)

^a^Laaveg and Ponseti’s functional rating scale [13]

^b^DSI [11, 12]

The mean pain score for all 13 feet was 1.8 (range 1–8); for the 11 fused feet, it was 1.2 (range 1–2). The two non-fused feet had scores of 2 and 8.

**Fig. 1:**
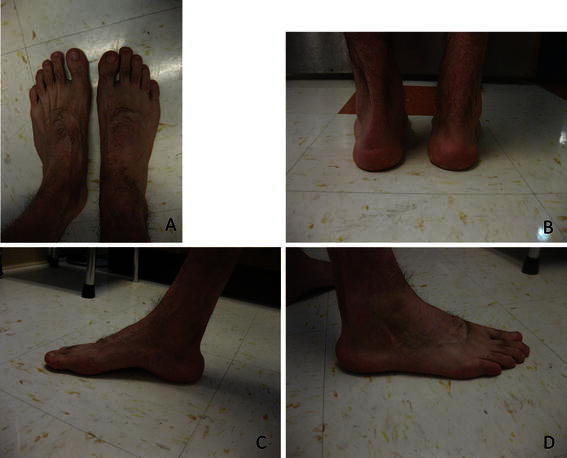
A 24-year-old male with right clubfoot who had soft tissue release at 6 months of age, and following recurrence had repeat soft tissue release with calcaneocuboid fusion at 6.3 years of age. Clinical photographs in standing: **a** anterior view, **b** posterior view, **c** medial view, and **d** lateral view

**Fig. 2:**
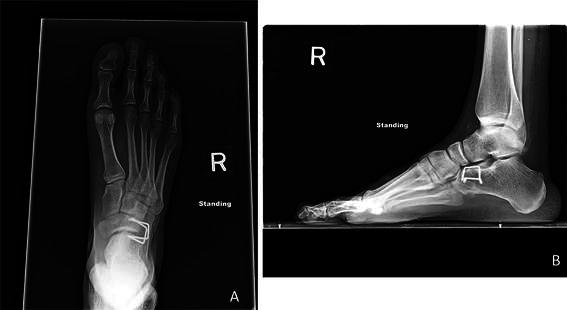
Weightbearing radiographs of right clubfoot in same patient as in Fig. 1: **a** anteroposterior view and **b** lateral view

For the clubfoot side of the six unilateral cases, the lower extremity was shorter by > 0.5 cm in only one patient (1.5 cm); calf circumference was smaller in all patients by a mean of 4.8 cm (range 2.6–6.2 cm), foot length was less in all patients by a mean of 2.6 cm (range 1.2–4.2 cm), and foot width was less in three patients with mean of 0.9 cm (range 0.5–1.4) and equal in the other three (Figs. 1, 2). For all 13 clubfeet, the mean maximum passive dorsiflexion was 3.7° (range −10 to 10°). For the 11 fused feet, the mean maximum dorsiflexion was 4.4° (range 0–10°).

## Discussion

The occurrence of stiff, relapsed or teratologic clubfeet has decreased in recent years, but remains ever present. This study is a unique 17-year follow-up of cases using the Dillwyn Evans procedure as a re-operation technique for failed clubfeet after initial posteromedial release. The results from this study showed that, while functional levels declined between 17-year and 5-year follow-up, patients overall had reasonably good function (AAOS score of 84.6), 61–76 % satisfaction with the current status of the foot, and low levels of pain (1.8). These findings are encouraging, given the many poor outcomes after repeat clubfoot surgery and limited arthrodesis of the foot [14].

Unfortunately, this study is limited in several ways from making a decisive comment about the durability of the Dillwyn Evans procedure as a salvage measure. The number of patients is small, the final age of the patients is relatively young (early to mid 20s), and there was a 50 % rate of lost to follow-up from the original cohort. The importance of follow-up was such that attempts were made on social media as well as internet searches, in addition to the latest contact information available in the chart [15]. However, it bears notice that the best treatment for this type of feet is still debated [16]. Recurrence rates after repeat soft tissue release have been reported to be 20–65 % [17, 18]. External fixation is effective, but may lead to generalized stiffness [14]. Osteotomies can considerably shorten the foot, and triple arthrodesis can theoretically cause overcorrection, as well as further increase the risk of arthritis development. Moreover, long-term follow-up data after revision surgery in recurrent clubfeet is scant.

There was a notable decline in outcome measures between the moderate and long-term follow-up groups from our institution (11/13 to 5/13 good or excellent results) [7, 19]. Many of the radiographs showed definitive signs of early sub-talar and tibiotalar arthritis. It is unclear what proportion of this progressive arthritis was due to the calcaneocuboid fusion performed at an early age, versus the repeated posteromedial releases. Because fusions around the talus are a known cause of pan-talar arthritis, and can lead to stiffness and decreased function, they are best reserved for difficult scenarios such as in recalcitrant foot deformities [20].

The results from this study can be compared with Graham and Dent’s 23-year follow-up of Evans’s patients from Cardiff, where the procedure was performed as primary surgery, as well as greater than 5-year follow-up of cases where the procedure was used in a revision setting (Table 4) [4, 6, 7]. Both groups had failures of the treatment, or patients who went on to triple arthrodesis: 11.5 % for the primary group and 2–5 % in the revision group. In our current study, no patients had yet gone on to require further surgery, though the declining scores point to it as a future possibility. The percentage of patients describing themselves as “satisfied” was 60–70 % in all series. In addition, two calcaneocuboid non-fusions occurred in feet that had undergone bilateral treatment. One foot had radiographic fusion while the other side had a stable pseudoarthrosis. The two patients had different outcome measurements, with one patient detecting no difference between his two feet and the other with a “fair” HJD FRS score on the non-fused side, versus a “good” rating on the fused foot. Other authors have reported an 84 % rate of radiographic fusion, with no evidence of any effect from bony fusion on function [4].

**Table 4:** Comparison of results from studies utilizing Dillwyn Evans procedure

	Current study	Original HJD study	Addison et al.	Graham and Dent
Mean length of follow-up (years)	17.5	5.5	9.75	23
Number of feet	13	27	45	60
Conversion to triple arthrodesis	0	1	1	7
Outcome	5 good/excellent	22 good/excellent	30 satisfactory	68 % satisfactory

Midfoot fusions in this skeletally immature group could theoretically lead to overcorrection of the deformity, although none of the current patients had midfoot overcorrection at the time of skeletal maturity. Additionally, none of the patients required any other procedures at the time of publication, including a triple arthrodesis, though functional scores were trending downwards. With regards to appearance of the foot, in our series 46 % described themselves as “satisfied”. There was no patient who presented with midfoot valgus. The average morphology score in the International Clubfoot score was 2.5 (range 0–7) out of 12. All patients could tolerate regular shoe wear and the average shortening was 2.6 cm. As comparison, Graham and Dent [4] had an average foot shortening of 1.6 cm and commented that “few of the feet looked absolutely normal: most were small with a broad forefoot, and 50 % had a widened heel.”

In summary, the Dillwyn Evans procedure remains worthwhile to consider as a possibility for stiff, recurrent clubfeet. A comparative study is required to distinguish whether it is a better treatment than the others available: external fixation, osteotomies, or soft tissue releases alone. It is difficult to separate ramifications of the revision posteromedial subtalar release from the calcaneocuboid fusion, but to the best of our knowledge, the calcaneocuboid fusion allowed for long-term maintenance of midfoot correction.
